# User-Centered Refinement of a Digital Tool for Tuberculosis Treatment Support: Iterative Mixed Methods Study

**DOI:** 10.2196/76742

**Published:** 2025-07-30

**Authors:** Sarah Iribarren, Omar Alfonso Aguilar Vidrio, Javier Roberti, Kyle Goodwin, Cristina Chirico, Hugo Telles, Barry Lutz, Fernanda Bornengo, Fernando Rubinstein

**Affiliations:** 1 Department of Biobehavioral Nursing and Health Informatics, University of Washington, Health Science Building, T602C, Box 357266, 1959 NE Pacific Street, Seattle, WA, 98195, United States, 1 2065435211; 2 Centre for Research in Epidemiology and Public Health (CIESP), Consejo Nacional de Investigaciones Científicas y Técnicas, Ravignani 2024, Buenos Aires, 1414, Argentina, 54 47778767; 3 Region Five, National Tuberculosis Control Program, Buenos Aires, Argentina; 4 Department of Bioengineering, University of Washington, Seattle, WA, United States; 5 Dr. Raul Vaccarezza Institute of Tisionemunology, University of Buenos Aires, Buenos Aires, Argentina; 6 Instituto de Efectividad Clínica y Sanitaria, Buenos Aires, Argentina

**Keywords:** tuberculosis, digital adherence support, human-centered design, mHealth, information systems research, mobile health, mobile phone

## Abstract

**Background:**

Despite the potential of digital adherence technologies to support patient-centered monitoring for tuberculosis (TB), there is limited research on incorporating indirect and direct adherence monitoring or assessing patients’ experiences with these technologies. The TB Treatment Support Tools (TB-TST) includes a comprehensive mobile app for patients and health care providers and a direct adherence metabolite test to report and monitor adherence.

**Objective:**

This paper describes the iterative refinement process of the TB-TST intervention.

**Methods:**

To refine the TB-TST intervention, we used an iterative approach involving multiple embedded mixed methods studies guided by the Information Systems Research framework and Design Thinking Process. Embedded studies included a randomized controlled pilot study, interviews, usability testing, and surveys with patients and experts to inform ongoing refinements. The project consisted of interface evaluation, high-level system design, and iterative redesign.

**Results:**

The TB-TST intervention was refined through 3 iterative phases. In Phase 1, based on feedback from pilot study participants and 4 experts in TB, improvements included an in-app discussion board, submission confirmations, and enhanced account recovery. Cultural adaptation was based on Hofstede’s dimensions. Phase 2 involved 4 Directed Research Groups and 19 stakeholders to redesign user flows, simplify reporting, and transition the app to a progressive web app, improving device compatibility. Phase 3 included usability testing cycles with 48 participants (26 patients and 22 health care professionals), yielding high satisfaction scores: patient app Mobile Health App Usability Questionnaire, mean 5.96 (SD 0.46); provider mobile dashboard IT Usability Evaluation Scale scores ranged from 5.83 to 6.23 out of 7, and optimization of interface and dashboard. Refinements included larger icons, streamlined onboarding, symptom summary enhancements, and a new cohort-level adherence graph. These modifications improved navigation, usability, and remote monitoring for patients with TB and providers in preparation for a multisite clinical trial.

**Conclusions:**

Combining multiple methods guided by the Information Systems Research framework and elements of the Design Thinking Process can help researchers and developers leverage the strengths of mixed methods iterative designs to create highly personalized and effective digital health interventions.

## Introduction

Tuberculosis (TB) is curable and preventable, yet in 2023, a total of 1.25 million people worldwide died, and 10.8 million people fell ill from TB [1,2]. Missing 10% or more of doses during TB therapy is associated with approximately six times increased risk of poor outcomes, including drug resistance and death [3,4]. Initiation and management of TB treatment in TB-endemic countries are challenging for both patients and providers, in part due to limited access to health care and a lack of patient-centered approaches. Traditional adherence monitoring strategies, such as directly observed therapy, self-reporting, pill counts, and refill tracking, are resource-intensive or produce indirect or inaccurate results that can lead to treatment gaps and failures [5-8].

In Argentina, TB remains a significant public health challenge. In 2023, the estimated TB incidence was 16,000 cases (35 per 100,000) [9]. TB incidence has increased by 37% since 2015, while deaths have risen by 3.6%. Alarmingly, treatment coverage was estimated at 87%, but treatment outcomes remain suboptimal, with success rates of only 52% for new or relapse cases and 42% for drug-resistant TB. Socioeconomic vulnerability is pronounced, with 48% of TB-affected households facing catastrophic costs [9]. Argentina faces persistent gaps in diagnosis, treatment outcomes, and support for TB-affected populations. TB treatment is provided free of charge and includes medication, routine clinical care, and laboratory tests. Most patients receive a 1-month supply of fixed-dose combination therapy, with a daily pill burden of 3‐4 tablets, and are asked to self-administer treatment with monthly follow-ups. There is no routine supervision between visits [10]. Despite free access to TB treatment, the lack of ongoing supervision and support contributes to poor adherence, undermining treatment success, and increasing the risk of resistance and transmission [11,12]. Addressing this adherence gap is critical to improving TB outcomes in Argentina and other high-burden settings.

Digital adherence technologies (DATs) have emerged as promising tools for enhancing medication adherence and monitoring during treatment [5,13,14]. DATs offer various monitoring approaches, including real-time tracking, personalized support, and direct communication between patients and health care providers [15]. Some DATs, such as video observed therapy, self-report via text, electronic pill bottles, and direct monitoring through embedded sensors and drug metabolite testing, have shown positive outcomes in TB treatment adherence [16-21]. Video observed therapy, a digital adherence strategy in which patients record or live-stream themselves taking medication, has demonstrated comparable treatment completion rates to in-person directly observed therapy and improved outcomes at reduced cost [12,22,23]. However, these technologies often rely on proxy indicators, such as video, self-report, or device openings, which do not confirm actual ingestion of medication [18]. Others have conducted spot urine drug metabolite testing at follow-up clinic visits [24]. Therefore, there is a growing need to incorporate objective, real-time measures of adherence that can enhance the accuracy of remote monitoring and enable tailored counseling and support based on verified nonadherence [21,25,26].

In response to this gap, we developed the TB Treatment Support Tools (TB-TST) intervention, a comprehensive approach incorporating indirect and direct adherence monitoring technologies for real-time, remote TB treatment monitoring and personalized support [27,28]. The TB-TST consists of a patient-facing mobile app, a health care provider dashboard, and a direct adherence metabolite test for adherence reporting and monitoring. Our team of experts in TB management, point-of-care diagnostics, and clinical informatics collaborated closely with patients and experts in Argentina, where the intervention was first developed and implemented. Argentina provides a relevant context for evaluating digital adherence innovations, given its growing mobile phone penetration, need for Spanish-language tools, and high rates of poor TB outcomes [29]. With this intervention, we aim to optimize TB management and support by providing an effective, accurate, adaptable, and scalable solution to empower patients, improve treatment adherence, enable patient-centered monitoring, and enhance health care provider reach. This study outlines the iterative refinement process of the TB-TST intervention prior to a multisite, randomized controlled clinical trial [30].

## Methods

### Study Design

**Figure 1.**
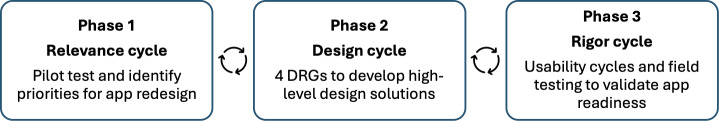
Simplified overview of the iterative design and refinement phases of the TB-TST intervention. DRG: Directed Research Group; TB-TST: tuberculosis Treatment Support Tools.

Our refinement process followed an iterative approach involving multiple embedded mixed methods studies, guided by an adapted Information Systems Research (ISR) framework, the Design Thinking Process, and human-centered design (HCD) principles (an approach that prioritizes the needs, preferences, and experiences of end users throughout the design and development process [31,32]. The ISR framework provides a structured approach that guides the development and evaluation of technology-based interventions through iterative cycles of relevance, design, and rigor cycles. The Design Thinking Process emphasizes empathy, ideation, and iterative prototyping to address complex problems. HCD prioritizes the needs, preferences, and experiences of end users throughout design and development. Embedded studies included a randomized controlled pilot study, interviews, usability testing, and surveys with patients and experts to inform ongoing refinements (Figure 1).

### Participants

Detailed recruitment processes and participant characteristics for each activity are outlined in their respective studies. This document summarizes the overall app refinement process and briefly describes the participant recruitment for each study or activity. For the pilot study, participants were recruited from Hospital Cetrángolo, a public hospital specializing in respiratory medicine in Argentina [10]. Eligible participants were adults (aged 18 or older) initiating treatment for drug-susceptible pulmonary TB, who had access to a smartphone and were able to use it themselves or with assistance from someone in their household. Informed consent was obtained in person prior to participation. Of 56 patients who began TB treatment during the recruitment period, 42 were enrolled and randomly assigned to either the intervention or control arm. The mean age of participants was 36.5 (SD 16.6) years, with equal representation of men and women. For the redesign cycles, TB experts and participants from pilot study provided feedback. For the usability testing cycles 1 and 2, participants were recruited via an email flyer using the University of Washington’s listserv for the School of Nursing and the School of Human Centered Design and Engineering, and from a web-based recruiting platform called Prolific Academic[33]. In usability testing cycle 3, the study was advertised via email flyers and posts on discussion boards for web-based TB communities, with additional participants being recruited from the UW listserv and Prolific Academic .

We conducted initial relevance, design, and rigor cycles to guide the first app version design [34, 27-29]. Refinement activities were informed by findings from the randomized controlled pilot study, feedback from patients and experts, HCD methods, and technical decision-making processes. Details of the study designs, primary aims, participant groups, data collection methods, and duration of each research activity are summarized in Table 1.

**Table 1.** Study activities, primary aims, participants, data collection, and duration.

Study or activity		Primary aims	Type of participants, n (%)		Data collected	Duration
Phase 1: Relevance or rigor cycle						
Pilot randomized controlled trial		Identify priority issues for refinement	Persons with TB^a^: 42 (91.3) Experts or providers: 4 (8.7)		App log data, two-way messages, exit survey, and interviews	11 months
Phase 2: Redesign cycle						
DRG^b^ 1: redesign patient interface		Map reporting flow, restructure feature layout, and assess cultural dimension	Stakeholders: 5 (100)		Stakeholder interviews with interface demonstration	10 weeks
DRG 2: refine patient interface		Refine primary features, including reporting and messaging, design, and onboarding	Persons with TB: 6 (66.7) Experts: 3 (33.3)		Feedback on designs	10 weeks
DRG 3: redesign provider interface		Redesign provider dashboard, define key functionality	Stakeholders: 5 (100)		Feedback on designs	10 weeks
DRG 4: refine provider interface		Refine provider mobile dashboard, assess feasibility	Stakeholders: 5 (100)		Feedback on designs	10 weeks
Phase 3: Rigor cycle						
Usability testing 1 (three iterative cycles)		Refine patient interface to improve accessibility and usability	Group of experts, persons with TB, and providers: 26 (100)		Talk-aloud sessions, MAUQ^c^ and Health ITUES^d^ surveys, and severity rating	20 weeks
Usability testing 2		Refine provider dashboard, identify usability, and accessibility issues	Providers: 6 (100)		Talk-aloud sessions, task success, and severity rating	10 weeks
Usability testing 3 (three iterative cycles)		Refine provider mobile-optimized dashboard and identify usability issues	Providers: 16 (100)		Talk-aloud sessions, MAUQ^c^ and Health ITUES^d^ surveys, and severity rating	10 weeks
Field testing with refined version		Prepare for trial, train new TSs^e^, and identify and address technical issues	Group of TSs^e^ and study team members: 10 (100)		Log of issues	3 weeks

a TB: tuberculosis.

b DRG: Directed Research Group.

c MAUQ: Mobile Health App Usability Questionnaire.

d ITUES: Information Technology Usability Evaluation Scale.

e TS: Treatment supporter.

### Phase 1: Relevance Cycle for App Interface Evaluation

The relevance cycle aimed to identify areas for improvement and establish refinement priorities based on the pilot results [32,35]. We analyzed pilot study participant exit interviews and surveys, stakeholder interviews, app use data, and digital messages[10,36]. The participants were followed for the full course of treatment (6 mo); intervention participants used app version 1.1 (2019) to self-report medication adherence, complete daily surveys, and submit photos of direct adherence tests [10,37]. The treatment supporters reviewed daily submissions on the provider-facing app and communicated with participants via WhatsApp to resolve questions and issues. The app’s source code has been published as a “release” on GitHub [38]. This phase identified core usability and cultural adaptation needs based on pilot findings, informing priorities for app redesign in subsequent phases. We also used Hofstede’s Cultural Dimension Assessment to inform development and increase usability based on Argentinian culture [39]. This assessment uses a framework for understanding cultural differences across countries based on key dimensions such as individualism, uncertainty avoidance, and power dynamics, which can inform the design of culturally appropriate interventions [39].

### Phase 2: Design Cycle for High-Level System Redesign

In this phase, we conducted four Directed Research Groups (DRGs) focused on aspects of the app redesign with multidisciplinary teams including experts in HCD, global health, and software engineering. DRGs are faculty- and research scientist–led, quarter-long opportunities for students to apply their skills in hands-on research projects. The first DRG addressed pilot-study-related issues, the second focused on redesigning the patient interface, and the third and fourth DRGs focused on refining the provider dashboard and interface, including designing a mobile-optimized provider dashboard. Designers established a clear visual hierarchy to present essential information consistently across app sections. We referenced the Web Content Accessibility Guidelines 2.0 standards (internationally recognized guidelines that ensure web content is accessible to people with disabilities, focusing on principles of perceivability, operability, understandability, and robustness) and conducted accessibility reviews to identify improvements for enhanced accessibility. Across the DRGs, we developed high-level plans and preliminary design refinements based on issues identified in Phase 1, including an information architecture diagram and low-fidelity design prototypes. We sought feedback on the redesigns from experts in TB, prior study participants, and clinical staff to assess if identified issues were addressed. This phase produced high-level design solutions through iterative prototyping and multidisciplinary collaboration, laying the foundation for a more accessible and user-friendly platform.

### Phase 3: Rigor Cycle for Iterative Redesign

Three usability testing cycles were conducted using a mixed methods approach, including think-aloud interviews and standardized usability surveys. Surveys included the Mobile Health App Usability Questionnaire—a validated tool used to assess the usability of mobile health apps, focusing on ease of use, interface design, and user satisfaction—and the Health IT Usability Evaluation Scale—a customizable instrument designed to evaluate the usability of health information technologies based on user perceptions of usefulness, ease of use, and impact on workflow [33]. A software developer built basic prototypes based on findings and feedback, with iterative improvements to the app interfaces made by the DRG and usability testing teams. This process continued until the team was satisfied with the app’s performance. Field testing over 3 weeks involved experts in TB and health care team members. Objectives included training treatment supporters on the TB-TST intervention, identifying technical issues, and preparing for the clinical trial. Treatment supporters and research team members acted as test users, reporting problems, raising questions, and interacting with treatment supporters. This phase validated usability and led to targeted final refinements and readiness for use in the next stage evaluation in a pragmatic clinical trial.

### Ethical Considerations

The pilot study (STUDY00007533) received approval from the Institutional Review Board at the University of Washington and the ethics committee of the research site. All participants provided informed consent prior to participating in the research activities. The trial was registered in ClinicalTrials.gov (ClinicalTrials.gov identifier: NCT03544476). The usability testing studies were deemed exempt from full Institutional Review Board review by the University of Washington Human Subjects Division under exemption categories: STUDY00014454 (Categories 2 and 101), STUDY00010640 (Category 2), and STUDY00003401 (Category 2). Ensuring patient privacy and ethical data use was a central consideration in the development of the TB-TST intervention, particularly given its implementation in low-resource settings. The app collects minimal personal information, limited to the user’s phone number, and the app is not publicly available via app stores. It is accessed only through an activation key provided by health care staff, and users must log in to enter the system. All participants underwent a comprehensive informed consent process covering both the digital platform and the urine-based metabolite test. Patients who participated in the pilot study and usability testing received compensation equivalent to US $25 for their time and contribution, in accordance with local ethical guidelines. Data transmission is encrypted, and the intervention was deployed in coordination with local health care teams to ensure compliance with ethical standards and data protection norms. Future iterations will continue to strengthen privacy safeguards and expand user education regarding digital data use.

## Results

### Overview

We present findings organized according to the three phases of the refinement process. Within each phase, we detail key design improvements informed by stakeholder feedback, usability testing, and technical considerations. A detailed mapping of research activities, findings, and corresponding redesign recommendations is provided Table S1 in Multimedia Appendix 1. Textbox 1 shows key improvements made throughout each phase. Table S2 in Multimedia Appendix 1 provides select screenshots of before and after refinement.

Textbox 1. **Main improvements in the three phases of the redesign process.**

**Phase 1: Pilot study and initial feedback**

- Integrated within-app discussion board
- Submission confirmation improvements
- Account recovery feature enhancements
- Security upgrades following the Open Web Application Security Project guidelines
- Cultural adaptations to improve clarity, structure, and motivation

**Phase 2: Redesign**

- Streamlined reporting paths and improved user flow
- Integration of progress tracking features
- Expanded monitoring functionality through notifications of low adherence risk for the provider dashboard
- Transition to a progressive web app to enhance device compatibility and reduce development time and costs
- Iterative improvements through Directed Research Group feedback cycles
- Symptom reporting modifications
- Layout and visual clarity enhancements
- Introduction of a mobile-optimized provider interface

**Phase 3: Usability testing and final refinements**

- Patient app refinements: improved readability, navigation, onboarding experience, and progress tracking
- Provider dashboard enhancements: zoom functionality, improved symptom summary views
- Mobile provider interface improvements: icon alignment, enhanced reporting navigation, and cohort-level adherence graph

### Phase 1: Pilot Study and Initial Feedback

#### Initial improvements

Pilot study participants and treatment supporters expressed overall satisfaction with the TB-TST app, rated it as easy to use, and indicated that they would recommend it to others beginning TB treatment. Access to a treatment supporter was consistently highlighted as a crucial feature supporting patient engagement and the TB treatment process. In addition to positive feedback, several technical challenges were identified, including slow loading of test images, intermittent internet connectivity, and difficulties initiating app use. In response, early modifications were implemented. An integrated within-app discussion board was developed to replace the initial system that required email login, addressing barriers for participants who did not use email. Submission confirmation mechanisms were enhanced, and network bandwidth efficiency was improved through code optimization, significantly reducing loading times. Improved account recovery features were introduced to mitigate problems arising from forgotten passwords or changes in device access. Security upgrades were guided by the Open Web Application Security Project top 10 standards, a globally recognized list of the most critical security risks to web apps, to guide secure software development, with system visualizations developed to facilitate detailed security reviews and protection against common vulnerabilities [40].

#### Assessment of Cultural Dimensions

Insights from Hofstede’s cultural dimensions assessment informed specific modifications to the app. Given Argentina’s high uncertainty avoidance, clear instructions for using the test strip, completing forms, and navigating treatment information were incorporated, alongside consistent use of imagery and terminology. To respond to the high individualism score, motivational materials encouraging consistent app use were integrated, and a walkthrough was developed to familiarize first-time users with the app’s features.

At the end of Phase 1, key usability and technical issues were identified and addressed through early design modifications and culturally informed adjustments to improve clarity, motivation, and user onboarding.

### Phase 2: Redesign and Platform Transition

#### User Flow

Building on Phase 1 feedback, the app’s user flows and information achitecture (IA) were redesigned to enhance usability. Daily reporting features were grouped prominently on the home screen, ensuring immediate visibility upon opening the app. Navigation paths were simplified by mapping more direct reporting routes and removing unnecessary options, such as the ability to jump between different parts of the report, which had previously caused confusion. Redundant steps were eliminated, and a progress page was integrated to enable users to conveniently review previous submissions, monitor adherence, and identify missed reporting days.

#### Platform Migration

We assessed platform performance in parallel, considering development time, user experience, and cost. Limitations associated with native app development, including device-specific constraints and extended development timelines, led us to explore using progressive web app (PWA) technology. A PWA is a type of web app that combines the features of mobile apps and websites, offering offline access, push notifications, and improved performance across devices without requiring installation from an app store. A simple prototype was developed to evaluate feasibility. Following piloting, we adopted the PWA approach for the TB-TST intervention. This transition enabled the integration of patient-provider communication features, supported the use of push notifications for medication reminders and treatment updates, and improved device compatibility across different operating systems. Adopting PWA technology also shortened development time and proved more cost-effective than native app development.

#### Iterative Improvements

Successive DRG cycles informed further refinement. Based on stakeholder feedback and DRG team recommendations, the symptom reporting layout was modified by removing emojis and incorporating submission confirmation prompts. Positive feedback led to the reinstatement of the color-coded calendar feature. Streamlining efforts focused on reducing unnecessary steps on the progress page and optimizing the home screen layout to reduce whitespace and prioritize critical actions. Requested actions for providers were positioned more prominently in the dashboard interface. A standardized design system was established to ensure consistency across the app. Additionally, a mobile-optimized version of the provider interface was introduced to improve user experience across devices and provide alternative and easy access. Redesigns were initially developed in English, and subsequent adaptations to Spanish carefully considered cultural and contextual differences.

At the end of Phase 2, significant enhancements were made to the app’s usability, navigation, and cross-platform performance. User flows and IA were streamlined to simplify reporting. The platform was successfully transitioned to a PWA to improve compatibility. Through iterative cycles, further refinements were introduced. These redesigns strengthened the app’s accessibility and responsiveness. Examples of key design changes between app versions 1.0 and 2.0 are presented in Table S2 in Multimedia Appendix 1.

### Phase 3: Usability Testing and Final Refinements

#### Patient App

Usability testing during the rigor cycle yielded high satisfaction scores for the patient app, with Mobile Health App Usability Questionnaire scores ranging from 5.2 to 6.9 and a mean of 5.96 (SD 0.46) on a 7-point scale. Several refinements were made based on user feedback. Font sizes were enlarged, and icons were modified to improve readability and meet user expectations. The treatment timeline was relocated to the home screen for easier access. Additional onboarding features were introduced, including a table of contents and the inclusion of diverse gender options. Navigation was improved by changing swipe directions and introducing a digital clock for logging treatment times. The progress bar was also redesigned to provide clearer feedback on adherence.

#### Provider Dashboard

Enhancements to the desktop provider dashboard are specifically targeted at improving the user experience for critical provider tasks. A zoom function was incorporated to facilitate closer examination of submitted photos, while confirmation dialogues with clear instructions were added to support the correct addition of new patients. A visible indicator was introduced for patients awaiting activation, and the calendar was repositioned for easier access. Additional viewing options for a patient’s symptom summary were provided, and the differentiation between one-to-one conversations and public discussions was refined for greater clarity.

#### Provider Interface

The mobile-optimized provider interface achieved consistently high usability scores across testing cycles (mean 6.21, SD 0.40; 5.83, SD 0.53; 6.23, SD 0.36, respectively). Refinements focused on aligning icons with user expectations, improving visibility across different screen sizes, and enhancing test strip tracking features. Reporting histories and recent activities were made more accessible, and alert flags were modified to display actual reporting percentages. Additional features were introduced, including a search bar for straightforward navigation, icon shortcuts, a patient filter option, and accommodation of varying device sizes. To support provider monitoring, a cohort-level graph of patient adherence over time was developed, offering a visual representation of engagement and progress.

At the end of Phase 3, usability testing showed high satisfaction with both the patient app and the mobile provider interface. Key refinements included improved readability, navigation, onboarding for patients, and enhanced functionality in the provider dashboard and mobile interface.

## Discussion

### Principal Findings

This study presents a comprehensive approach to guide the TB-TST intervention’s iterative redesign and refinement process. Most TB-related apps target health care workers or provide general TB information, with few focusing on patients and none supporting patient self-tracking or side effects monitoring [22]. Consequently, this study represents a significant advance in combining a patient-focused support app with a direct drug adherence test, leading to an enhanced digital adherence technology tool to improve TB treatment outcomes. Moreover, the app’s support for multiple languages is a notable strength, as non-English options for TB-related apps are limited in the marketplace [22].

### Interpretation of Findings

While the ISR framework is typically applied during the initial development phase, we also used it later in this study to guide iterative refinement. To enhance the app’s design, we developed meta-artifacts, such as app flow diagrams, IA, and feature outlines, which were instrumental in re-evaluating and optimizing the placement of various features. In parallel, we considered how the tool could function effectively across diverse global health contexts [31]. Although a common criticism of the ISR framework is that it may privilege researchers’ ideas over user perspectives, our integration of HCD and engineering approaches prioritized user-centeredness, ensuring that the voices of people using the system in real-world environments remained central. Throughout the design process, we consistently aimed to ensure that the app’s development was driven by the needs, preferences, and lived experiences of end users.

### Integration of Feedback and Refinement

Although conducting a randomized controlled pilot study for testing may not be feasible during every design cycle, it was instrumental in gathering valuable user feedback on the app and tools and in assessing the initial impact on treatment outcomes. Following the pilot study, our focus shifted toward addressing critical issues and integrating the needs and preferences of patients and treatment supporters into the app redesign process [41]. We systematically addressed the problems identified and incorporated user feedback into subsequent iterations. Research activities included evaluating app utilization, conducting interviews, analyzing patient-provider communications, and undertaking usability testing. Our HCD approach prioritized ease of use, considering challenges such as limited internet access and varying levels of technological literacy among users. In response to the feedback gathered, we introduced new features to enhance the overall patient treatment experience and to align with World Health Organization guidelines for digital health interventions [42]. This iterative approach enabled continuous refinement of the TB-TST intervention, ensuring it meets the unique needs of patients and health care providers across diverse settings.

### Implementation Considerations

The use of targeted DRGs proved to be an effective strategy for guiding research activities. This approach enabled us to draw on diverse perspectives while redesigning the patient app and provider dashboard interfaces. We streamlined these activities to increase efficiency. Open communication channels among stakeholder groups were fostered to optimize processes and facilitate discussions on emerging design prototypes and research findings. We developed onboarding materials to train new team members rapidly and used project management methods to plan weekly activities and target specific outputs. Engaging stakeholders and incorporating qualitative and quantitative feedback is crucial to developing valuable and widely adoptable mobile health apps [43]. We recommend maintaining a consistent core team across cycles, with new members shadowing experienced colleagues before joining a DRG. Establishing clear project objectives and priorities at the outset of each design cycle is essential to managing scope creep [35]. Regular reviews and evaluations of design features, conducted through weekly or biweekly meetings, help ensure alignment with project and user goals.

### Strengths and Limitations

A commonly cited limitation in digital interventions is their dependence on smartphones, internet access, and digital literacy, which may restrict their usability in low-resource or rural settings—often those most in need of adherence support. However, this concern was proactively addressed through offline functionality, plain language, and supportive videos. Although the original concept was SMS text messaging–based, formative research revealed that WhatsApp had become the predominant communication platform in the study context, prompting a shift in design. During the pilot study, only two participants were excluded due to a lack of mobile phone or internet access. Despite a few individuals being unable to participate due to phone access, it is still critical to incorporate flexible implementation strategies and ensure that future iterations of the intervention are inclusive of users with varying levels of technological access. Although all pilot study participants were invited to in-person exit interviews, not all attended, potentially omitting valuable insights. Re-engaging participants for subsequent iteration evaluations also proved challenging. To mitigate these issues, we conducted supplementary interviews and usability testing with individuals familiar with regular medication use or respiratory conditions. Qualitative findings from the design process highlighted the central role of treatment supporters’ accompaniment and the value of the two-way communication and self-monitoring features. The metabolite test primarily functioned as a prompt for enhanced monitoring and supportive conversations when adherence concerns arose.

### Future Directions

Structured usability studies are ongoing to support further app refinement. Lessons from this iterative process have been incorporated into smaller refinement cycles, with all design changes and underlying rationales rigorously documented to enhance transparency and track the app’s development. While this study focused on the iterative design and refinement of the TB-TST intervention, we will evaluate and report the combined and individual contributions of its core components.

### Conclusions

This study demonstrates the value of combining multiple embedded mixed methods, guided by the ISR framework and elements of the Design Thinking Process, to refine the TB-TST intervention. Feedback from diverse stakeholders, including experts of TB, patients with TB, and HCD specialists, was instrumental in enhancing usability and addressing key issues. Our engagement with DRGs validated their role in strengthening refinement processes, advancing knowledge, and creating meaningful research opportunities for skilled students. The framework presented here offers a practical guide for future digital health research, highlighting the importance of iterative design in developing effective and innovative interventions. Embracing such approaches can enable researchers and developers to harness the strengths of mixed methods designs to improve health care interventions.

## Supplementary material

10.2196/76742 Multimedia Appendix 1 Supplementary tables.

## Abbreviations

DRG: Directed Research Group
HCD: human-centered design
ISR: Information Systems Research
PWA: progressive web app
TB: tuberculosis
TB-TST: TB Treatment Support Tools
