# Supplementary material for: User-Centered Refinement of a Digital Tool for Tuberculosis Treatment Support: Iterative Mixed Methods Study
Source: J Med Internet Res. 2025 Jul 30;27:e76742. doi: 10.2196/76742 (PMC12309858; doi:10.2196/76742)
Supplement: Multimedia Appendix 1 [file jmir-v27-e76742-s001.docx]

**Table S1. Research activities, main findings and redesign recommendations**

| **Research Activity** | **Main Findings** | **Redesign Recommendations** |
| --- | --- | --- |
| **Evaluation of the current state of app interfaces and Additional User Research** | | |
| Pilot study exit interviews | Connectivity issues  Not all app features were used (e.g., notes)  Need an onboarding tour of the app’s features  Request for medication reminders | Build offline capability  Add onboarding walkthrough for primary features  Add daily medication reminders, and inquiry-based on missed reports |
| TB experts Interviews | The discussion forum was considered an important feature to maintain but needed to be modified to clarify use cases  Group chat messaging promoted communication among participants  Some participants reported symptoms outside of the app (messaged coordinator directly) | Change forum to group chat discussion  Increase the functionality of in-app messaging and announcements  Improve confirmation feedback for test strip receipt and review  Simplify side effect reporting flow |
| Interactive messages analysis | Higher rates of messaging during the first two months of treatment  Patients wanted quick responses  Inconsistent positive reinforcement and milestone-related messages across participants and over time  Treatment progress updates were important for participants and treatment supporter  Assistance is needed to regain access to the app  Test strip results were unclear to participants, and unclear test results prompted treatment supporters to inquire about how the test was done | Patient interface:  Onboarding could reduce initial questions  Feedback for successfully submitted report  Addition of treatment progress timeline visual Motivational notifications of treatment milestones to alert participant of their progress  Timely treatment messages  Treatment supporter interface:  Capture more data about possible reasons for low adherence  UI elements to prompt interactions  Identify app usage data to inform evaluation  Build a data dashboard for an overview of the cohort  Automatic missed treatment inquiry |
| Critical Assessment of Technical Limitations | The application was slow to load data for users  System logging is extensive  Invalid data was common  The software system was complicated to extend (e.g., translations not standardised, tightly coupled architecture)  The low resolution of some test strip photos  Patient Reporting timeline was jumbled | Reorganize Data Model  Use modern browser APIs  Standardise Internationalisation  Move forward with progressive web application system rebuild |
| Hofstede’s Cultural Dimension research for Argentina | High score for “Uncertainty Avoidance,” (measures how much the members of a culture feel threatened by ambiguous or unknown situations)  A high score on “Individualism” (reflects the degree of interdependence a society maintains among its members)  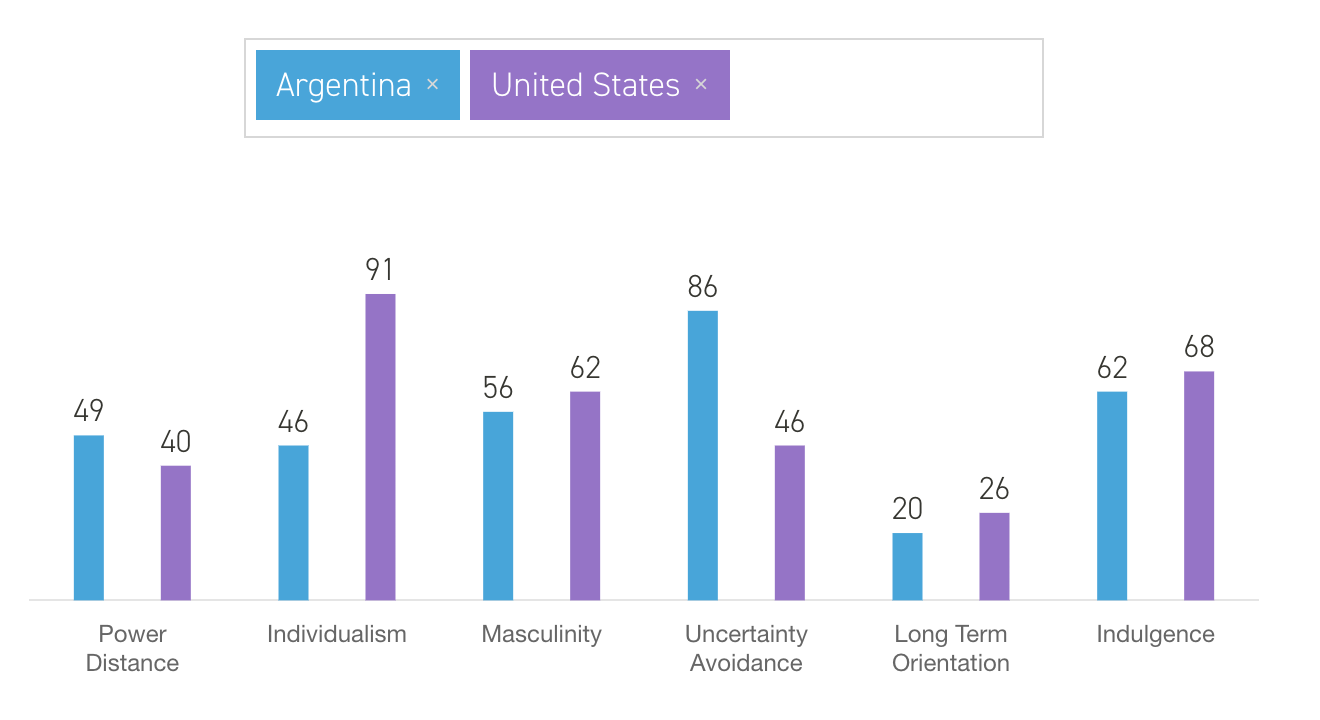 | App walkthrough/tutorial to familiarise users  Clear instructions on test strip and image  Use of consistent icons, text, and design elements throughout the app  Allow users to take control over their actions within the app  Create patient discussions to allow for interpatient communication and support |
| **Planning / High-Level System Design** | | |
| Application Platform evaluation | Progressive Web App Benefits  Cross-platform  It does not require adding new programming languages  Easy installation process for users, easy updates  Offline capabilities  Potential for Notifications / Reminders  Improved APIs for camera access  Native Application Drawbacks  Cross-platform solutions lead to large application sizes and slow downloads.  App store approval processes.  Less active development community, reliance on packages developed by for-profit organisations.  The updating process is more complicated | Advance with progressive web app development |
| System diagram and data modelling | Graphic visualisation of the system and data model developed ‘At a glance’ | Recommendations to refine the technical system |
| Information Architecture | Placement of some in-app features not intuitive  Navigation could be improved  Some users were unaware of features other than the required daily actions | Reorganisation of features into four tabs with well-defined purposes |
| Flow diagrams | Needed to accommodate the documented reporting use cases from a pilot study  Many completed the components of their daily reports at different times.  Internet connectivity issues often force users to submit multiple days’ worth of reports at a single time.  It is challenging to know if a step in their report has been completed and/or saved | Limited the screens required for each of the daily report’s components to two.  Allow for daily report components to be completed separately, i.e., at different times  Allow for three days of reports to be submitted retroactively  Explicit signalling that a step in the reporting flow was completed and saved |
| Feature outlining | App version 1.0 had a calendar on the home page that served as an access point for submitting reports, viewing previously submitted reports, and an overview of the treatment progress.  Other app features, “notes” and “progress”, did not have well-defined purposes | Defined each feature's purpose before visual design and prototyping to help in refinement |
| **Iterating Application Design *(see Appendix 1 for screenshots of before and after)** | | |
| Weekly design sprints | Weekly presentation of design iterations, discussions, and summary of next steps | Low- and high-fidelity prototypes produced  Interface design decisions made  Preparation for patient and treatment supporter evaluation (e.g., list of questions) |
| System evaluation | Restricted access to sign up for account needed  Phone numbers were unverified and could be improperly formatted or false.  Browsers cannot store API access credentials securely | Require new users to submit an activation code generated by a treatment supporter  Move to http-only cookie-based authorisation system (recommended by OWASP) |
| Feedback on minimum viable products | Some Android phones back cause apps to close, leading to a bad user experience.  Medication reminder setting hard to find  The settings button was not intuitive.  The information section was too wordy.  Severe symptoms sections might scare users from reporting.  Users might report inaccurate data if allowed to edit old reports  Issues with application loading on iOS  Fonts not loading properly  Some styles were broken | Refactor code to store some UI state in URL, allowing the back button to function.  Extend sessions so users are not logged out when the app is closed.  Move the location of the medication reminder option  Change settings logo  Reduce information section content and add informational video options  Limit prior day reporting to 3 days  Improve iOS compatibility |

**Table S2. Screenshots/artifacts of prior app and refined version based on research results and main problems and solutions addressed**

| **Version 1.1** | **Version 2.0** |
| --- | --- |
| **Home Page** | |
| **Problems:**  Unclear if the treatment log for the day is complete  Left opportunity to submit multiple reports if they thought that it was incomplete  Calendar should start on Sunday.  No way to take photo separately from report  No personalized messages about treatment  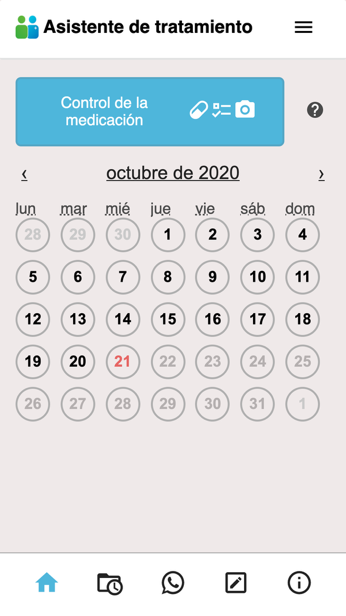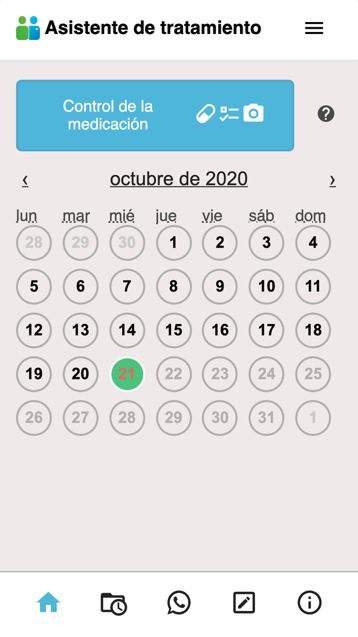 | **Solutions:**  Clear confirmation that report was submitted for the day  Added greeting for personalization  Simplified view of progress on home page (streak, visual, and timeline)  Set medication reminders and add appointments  Settings  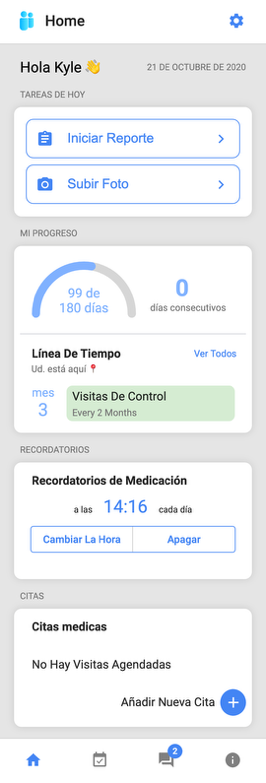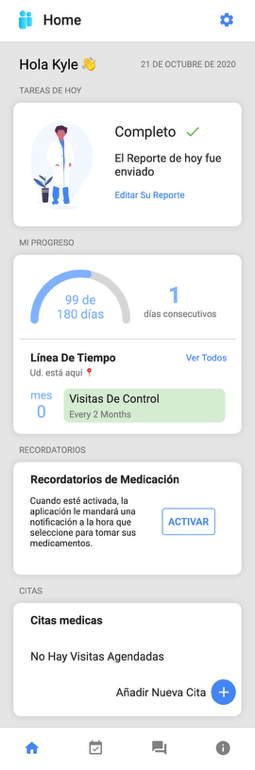 |

| **Report tracking /Calendar** |  |
| --- | --- |
| **Problems**  List format/drop down of all reports  Reporting data was organized by step instead of by day  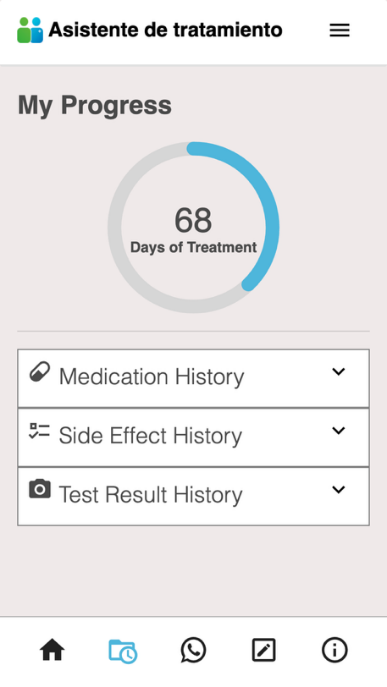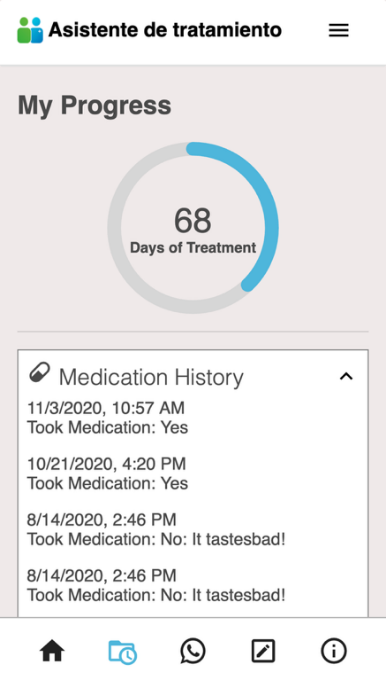 | **Solutions**  Calendar of all report events with added key for each state  Submit missed reports  View details of each report  Moved the progress bar visual to home screen for emphasis  Shows a continuous “streak” to motivate patients  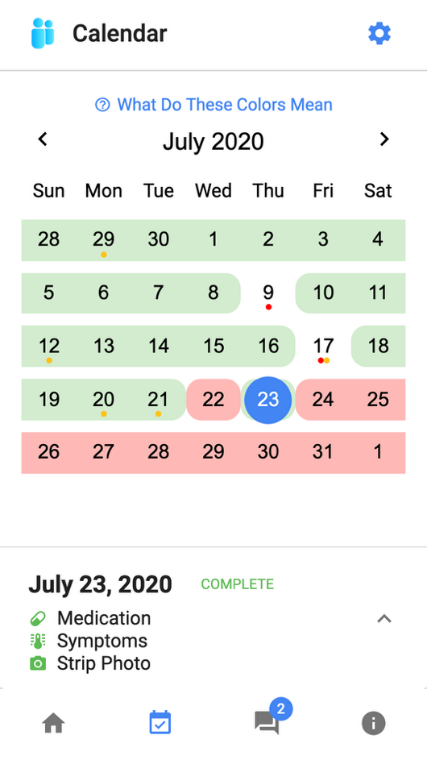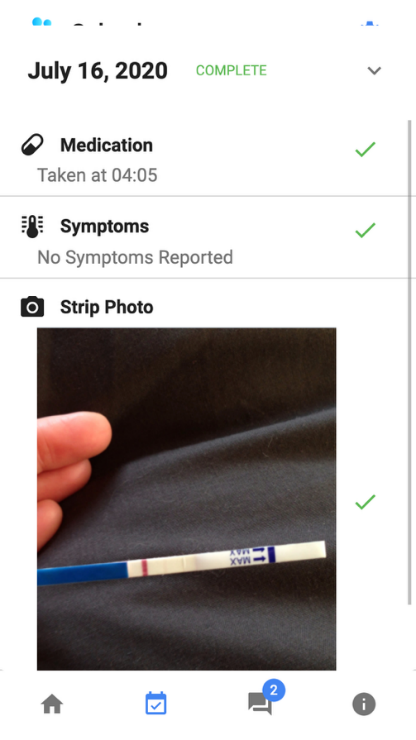 |

| **Notes** | |
| --- | --- |
| **Problems:**  Not utilized by pilot study participants.  No clear purpose for notes.  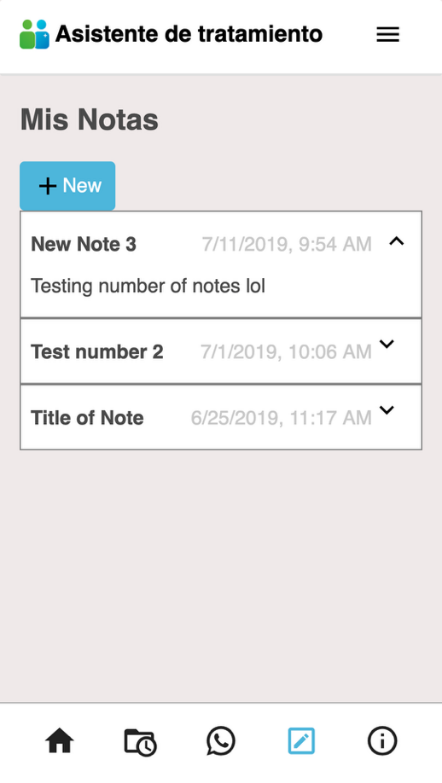 | **Solutions:**  Add appointment times  Able to add in notes for appointments  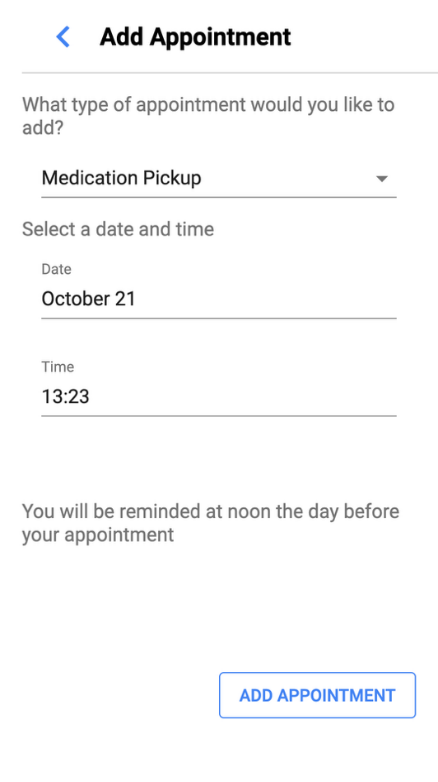 |
|  |  |

| **Communication/messaging** | |
| --- | --- |
| **Problems:**  Lack of notifications made it difficult for patients to have conversations in the discussion boards.  Patients were unfamiliar with discussion forums compared to WhatsApp style chat.  WhatsApp app was the only way for patients to contact their coordinators.  Difficult to identify who sent which messages because they were all formatted the same way.  Coordinators could not delete (i.e., hide from other patients) inappropriate messages.  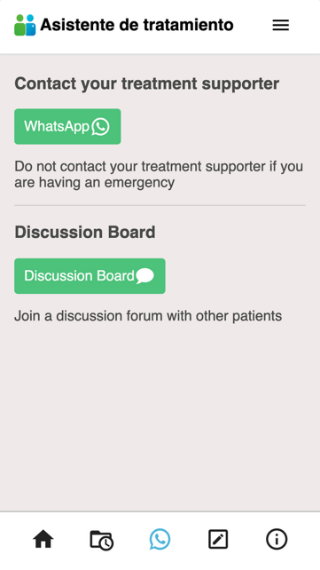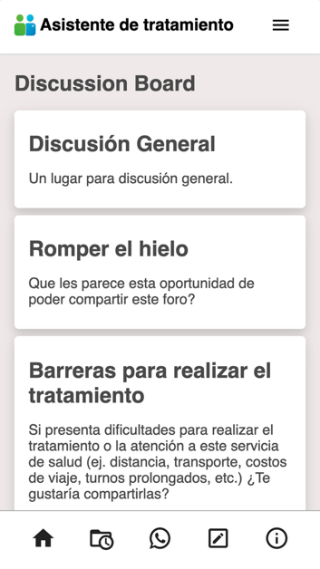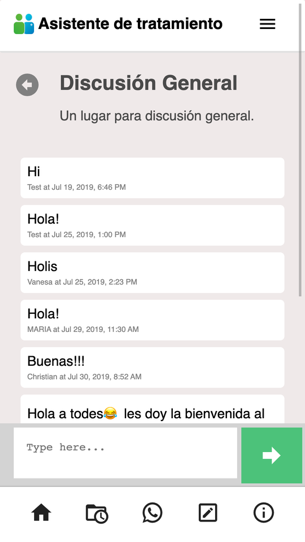 | **Solutions:**  Added notifications to allow for real-time conversations.  No additional information is required for patients to participate in the group discussions.  Transition to similar chat format of WhatsApp which is readily recognizable by most patients.  Created private chats for patients to contact their coordinators.  Coordinators can hide inappropriate messages from other patients.  Photos can be sent for more in depth help with treatment issues  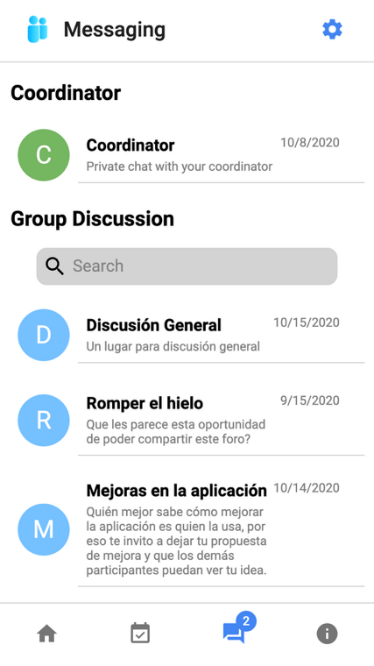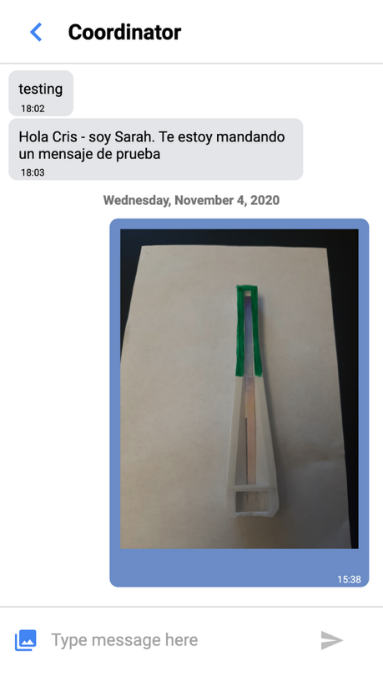 |
|  |  |

| **Information Page** | |
| --- | --- |
| **Problems**  Long list of information.  Videos linked inside the app resulted in small screen view.  Only included information about TB and treatment.  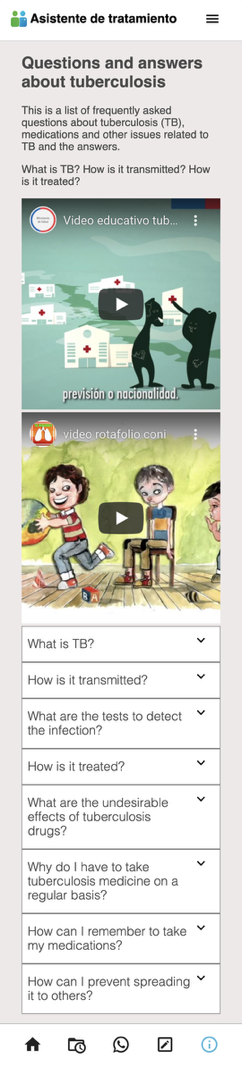 | **Solutions:**  More support added for app use (e.g., videos on how to submit a report, conduct test, app walkthrough).  More sections added to address information needs  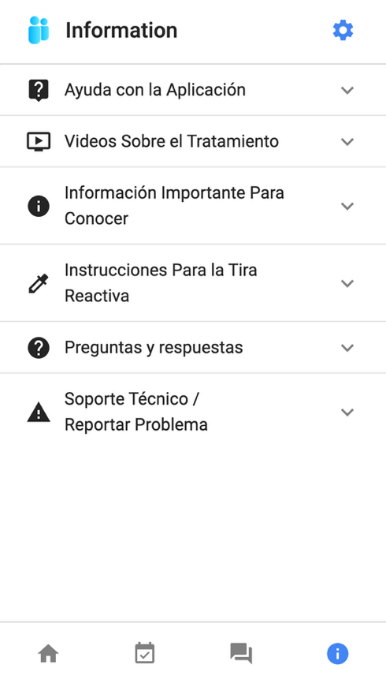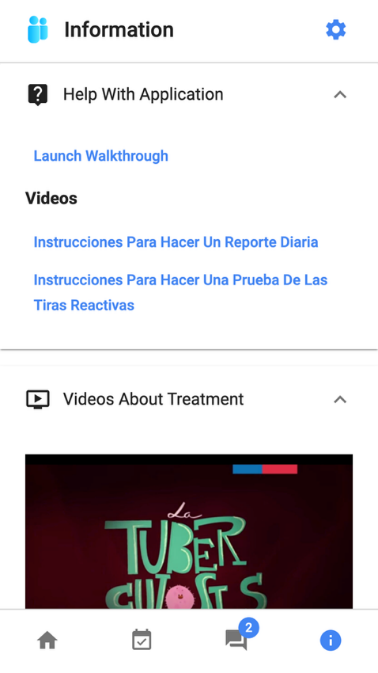 |

| **Reporting Sequence** | |
| --- | --- |
| **Problems:**  Difficult to identify which steps had been completed.  Steps could be completed in any order.  Test strip photos were required for each day.  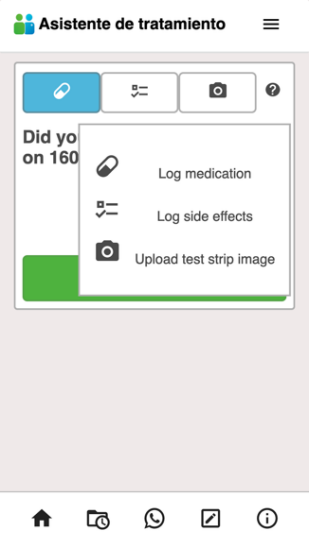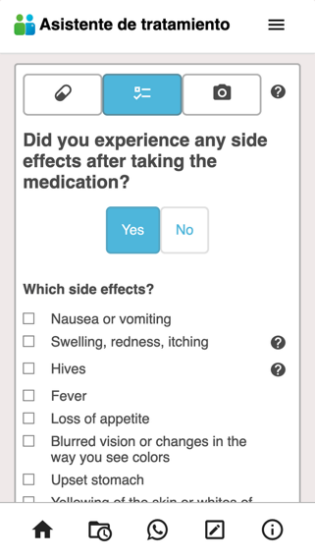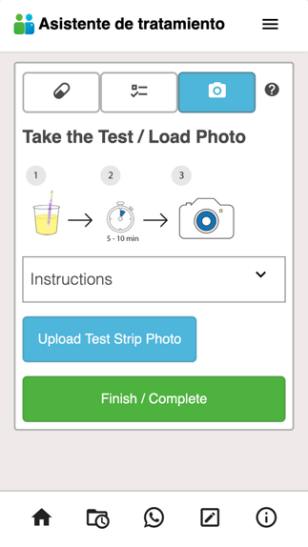 | **Solutions:**  App saves progress and opens to last completed step.  Steps must be completed in same order every time.  Added new reporting question for overall wellbeing  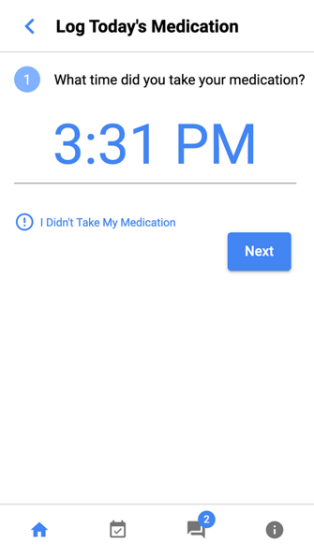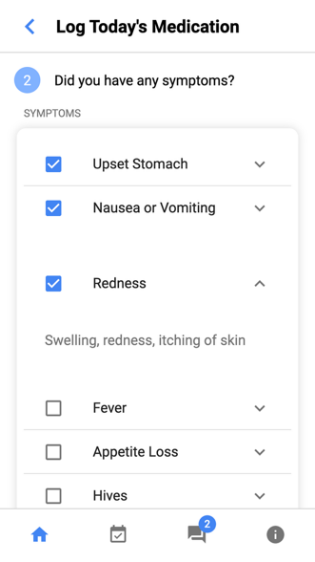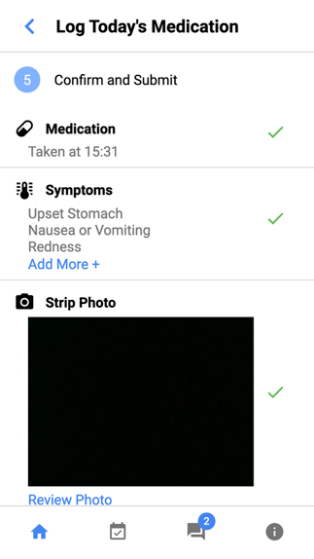 |

| **Onboarding/App Help** | |
| --- | --- |
| **Problem:**  Patients required help using the application during their first few weeks of treatment  Sometimes they would forget about  Many patients were seeking help for similar issues | **Solutions:**  Added Interactive walkthrough to support first time users and minimize initial messaging required to understand how to submit a report and use each app feature  Added explanations for visual representation of reports  Produced video walkthroughs to mitigate common issues and questions  Instruction in written and visual form for how to take the test and upload an image  Added table of contents for easier access  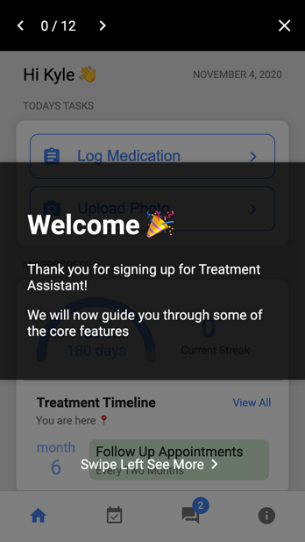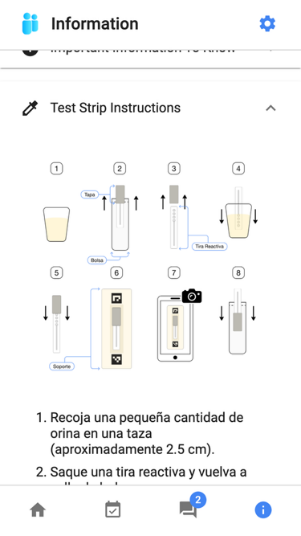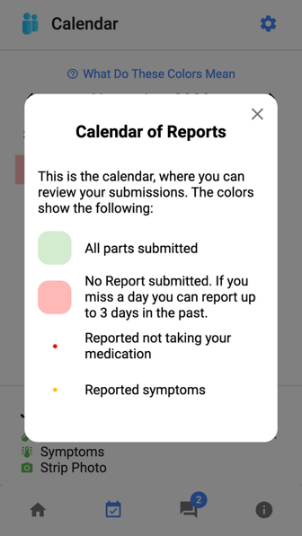 |
